# Use of ubiquitous chromatin opening elements (UCOE) as tools to maintain transgene expression in biotechnology

**DOI:** 10.1016/j.csbj.2022.11.059

**Published:** 2022-12-07

**Authors:** Rebecca E. Sizer, Robert J. White

**Affiliations:** Department of Biology, The University of York, Heslington Road, YO10 5DD, UK

**Keywords:** Barrier, CHO cell, Epigenetic silencing, Industrial biotechnology, Insulator, Ubiquitous chromatin opening element

## Abstract

Amongst the most important outputs of the biopharmaceutical industry are recombinant proteins, many of which are produced by integrating transgenes into the genomes of mammalian cells. However, expression is highly variable and can be unstable during prolonged culture. This is often due to epigenetic mechanisms silencing the transgenes. To combat this problem, vectors have been engineered to include ubiquitous chromatin opening elements (UCOEs) that protect against silencing. Here, we recount the evidence that UCOEs can modify chromatin environments and benefit biomanufacturing.

## Highlights

• A major challenge for industrial biotechnology is to maintain transgene expression.
• Transcription can be insulated against epigenetic silencing using UCOEs.
• This review describes key features of UCOEs and their performance in contexts relevant to industry.

## 1 Introduction

A major ongoing challenge for the biopharmaceutical industry is that expression of recombinant proteins by mammalian cell lines is highly variable and often unstable during prolonged culture [1], [2], [3], [4]. This production instability has been observed in up to 63 % of all recombinant Chinese hamster ovary (CHO) cell lines and is highly unpredictable, requiring lengthy stability studies, which are labour-intensive and costly [5], [6]. As CHO cells are the most widely used mammalian platform, providing 84 % of monoclonal antibodies in 2018 [7], the impact of this problem is substantial.

Epigenetic silencing of the recombinant gene of interest (GOI) is a major contributor to productivity loss over time [3], [8], [9], [10], [11], [12], [13], [14], [15]. This involves non-mutational gene inactivation through repressive covalent modifications, including methylation of cytosine in DNA and posttranslational modifications of histones, which can act independently or together to determine the transcriptional state of a gene [16], [17], [18], [19]. Many studies have highlighted aberrant DNA methylation and hypoacetylation of histone H3 as contributing to variegated transgene expression or complete silencing over time [3], [9], [10], [13], [20], [21].

Production instability caused by epigenetic silencing can be ameliorated by genetic elements, known as insulators, which can stop the spread of repressive chromatin. Multiple types of gene regulatory elements have been tested for their ability to sustain transgene expression, including ubiquitous chromatin opening elements (UCOE), stabilising anti-repressor elements (STAR), Scaffold or matrix attachment regions (S/MAR) and the DNase I hypersensitive site 4 insulator of the chicken beta-globin locus control region (cHS4) [22], [23], [24], [25], [26], [27], [28]). When these were tested in parallel, a UCOE improved transgene expression to a greater extent than any other genetic element tested in this context [29]. Accordingly, UCOEs have become popular for biomanufacturing applications, as a way to reduce production instability [30]. In this review, we explore the effect of UCOEs on their surrounding epigenetic environment and discuss their potential to ameliorate epigenetic silencing in the context of biomanufacturing.

## 2 Discovery of UCOEs

**Fig. 1:**
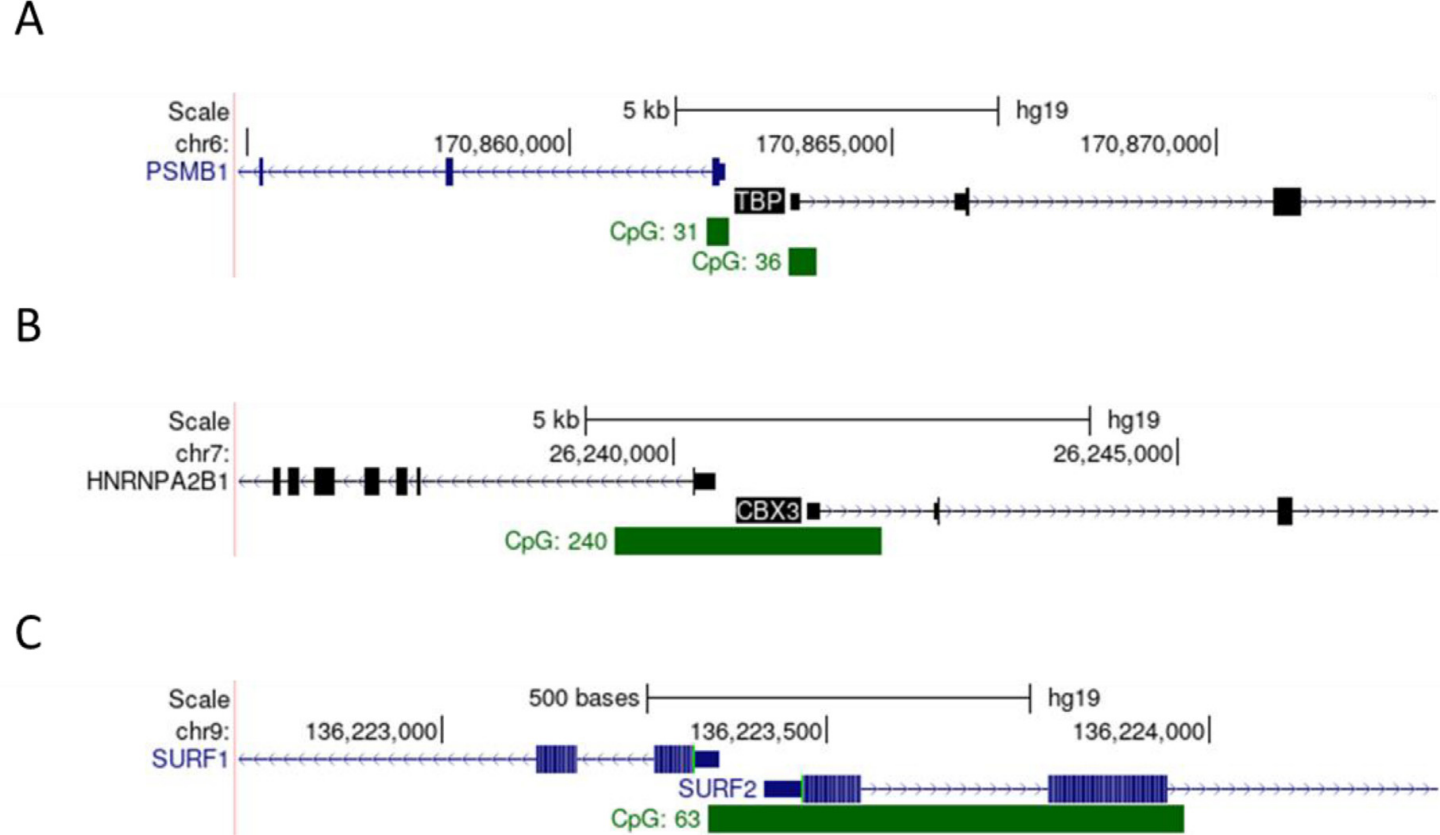
UCOES are characterised by a CpG island. Screenshots of the UCSC genome browser showing the location of the CpG island at the (A) TBP/PSMB1 UCOE, the (B) A2UCOE, and the (C) SRF-UCOE.

Four fragments of DNA have been classified as UCOEs to date: three human (TBP/PSMB1, HNRPA2B1/CBX3 (dubbed A2UCOE) and SURF1/SURF2 UCOEs) and the murine Rps3 UCOE. Of these, the first to be described as a ubiquitous chromatin opening element was the human TBP/PSMB1 fragment; this contains three genes within a ∼ 50 kb window, of which two housekeeping genes, encoding TATA-binding protein (TBP) and the proteasomal subunit C5 (PSMB1), are divergently transcribed from promoters spaced ∼ 1 kb apart (Fig. 1A). The presence of a DNase I hypersensitive site at the TBP and PSMB1 promoters in cells provides evidence of accessibility and suggests that the region contains regulatory elements that promote an open chromatin conformation. This was supported by expression of exogenous TBP over 60 days after random integration of a 44 kb sub fragment into murine fibroblasts, suggesting that this UCOE can protect against silencing activities from nearby repressed regions [31].

Like the TBP/PSMB1 UCOE, the A2UCOE also contains three genes, two of which are divergently transcribed housekeeping genes, encoding heterologous nuclear riboprotein A2/B1 (HNRPA2B1) and heterochromatin protein 1Hs-y (CBX3) (Fig. 1B). Both the TBP/PSMB1 and the A2UCOE dual promoter regions are encompassed by extensive, methylation-free CpG islands, of 1.96 kb and 3 kb, respectively [32]. It was proposed that the high content of unmethylated CG dinucleotides may be inherently able to open the chromatin and promote transcription [33]. Multiple shorter fragments of the A2UCOE retain anti-silencing properties, the smallest consisting of a minimal 0.7 kb fragment lacking the HNRPA2B1 promoter [34], [35].

Rudina and Smolke [36] screened the human genome using an algorithm designed to identify regions with similar characteristics to established UCOEs; they searched for regions that (1) are located close to a housekeeping gene promoter, (2) have at least a 50 % overlap with a CpG island, (3) are marked with euchromatic histone modifications and (4) have a binding site for CTCF, a transcription factor with well-recognized insulator function [37]. Candidate UCOEs were cloned upstream of a reporter gene and transfected into P19 cells to assess their effectiveness [36]. Of 8 candidates tested, the most successful prevented silencing of the reporter over 19 days, irrespective of its orientation; it was named SRF-UCOE, as it encompasses the divergently transcribed promoters of the SURF1 and SURF2 genes (Fig. 1C). Excluding the intergenic region between these divergent promoters ablated insulation in three out of four reporter constructs tested, each with a different transgene promoter; thus, the divergently transcribed promoters and the intergenic region contribute to the insulator activity of the SRF-UCOE in this context. Despite being identified by the algorithm, three candidates failed to demonstrate insulator activity, indicating that the criteria used in this study are insufficient to identify UCOEs reliably.

The mouse Rps3 UCOE has a single gene promoter, unlike the three human UCOEs, which all contain two divergent promoters. Divergent transcription may therefore not be a requirement for UCOE function. The Rps3 UCOE fragment is 3.2 kb in length and includes a comparatively small CpG island of 358 bp; nevertheless, it was able to provide high and homogenous eGFP expression over 28 days of continuous culture in CHO-K1 cells [38], [39]. These early studies described the first characterisations of UCOEs and highlighted their potential to counteract epigenetic silencing.

## 3 The epigenetic signature of UCOEs

**Fig. 2:**
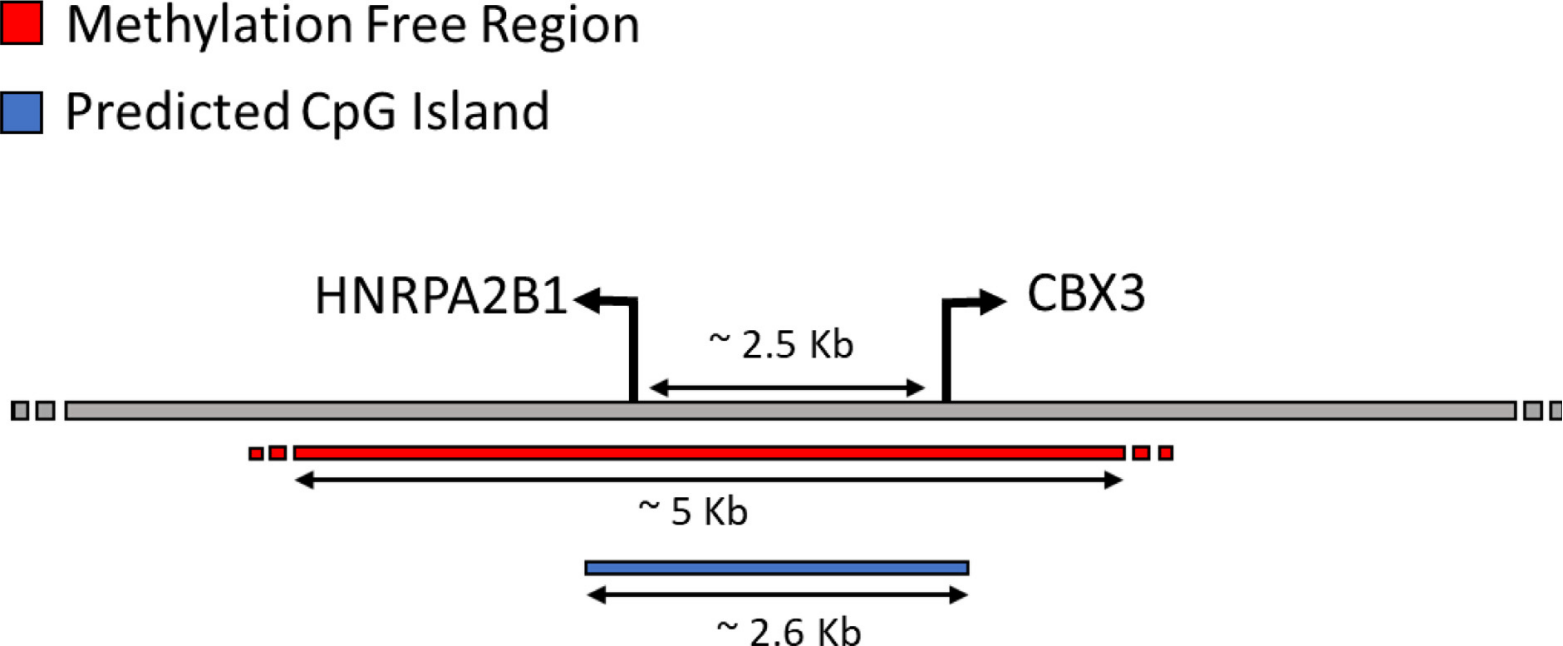
The predicted CpG island and actual methylation free region at the A2UCOE in Human PBMC DNA.

The epigenetic signature of UCOEs was first investigated at the endogenous A2UCOE locus in human peripheral blood mononuclear cells. A 2,644 bp CpG island spans the divergently transcribed promoters of HNRPA2B1 and CBX3. Bisulfite conversion confirmed a ∼ 5 kb methylation-free region that extends beyond the CpG island from ∼ 3 kb downstream of the HNRPA2B1 promoter to 1.5 kb into the CBX3 gene (Fig. 2) [40]. Native chromatin immunoprecipitation revealed that the HNRPA2B1 and CBX3 promoters are enriched for euchromatic histone marks, including acetylation of histones H3 and H4, but are depleted of the heterochromatic mark H3K27me3. Acetylation of histone H4 remains high throughout this locus even if the adjacent chromatin has DNA methylation [40]. This extensive euchromatic region with low repressive marks was proposed to facilitate expression of linked genes by promoting a transcriptionally-permissive environment.

A 1.2 kb A2UCOE fragment maintained its endogenous epigenetic characteristics at an ectopic genomic location in P19 cells [41]. Indeed, when linked upstream of a spleen focus forming virus (SFFV) promoter driving an eGFP reporter, the A2UCOE resulted in a marked reduction in SFFV DNA methylation, which correlated with ∼ 8-fold increased stability in eGFP expression over 17 days [41], suggesting that the A2UCOE can stabilise expression of linked genes at exogenous locations by, at least in part, resisting DNA methylation. A core sub fragment of the A2UCOE from the first two exons of the CBX3 gene caused profound enrichment of H3K4me3 at a linked SFFV promoter, as well as a decrease in the heterochromatic marks H3K27me3 and H3K9me3; the transcriptionally active form of RNA polymerase II was also detected [34]. These findings support the model that UCOEs at exogenous sites can generate open chromatin environments at linked promoters and resist silencing. When integrated adjacent to a heterochromatic telomeric locus in HeLa cells, a relatively short A2UCOE fragment was reported to deplete the surrounding chromatin of heterochromatic histone marks [42]. However, one of two reporters was not expressed, despite hyperacetylation of histone H3 [40], [42], indicating that this is insufficient for activity.

The ability of A2UCOE to resist epigenetic silencing depends on the promoter it is linked to. Alongside a tissue-specific myeloid MRP8 promoter, the HNRPA2B1 region of A2UCOE became significantly methylated after 21 days in P19 cells, as did 87 % of CpG dinucleotides in the linked MRP8 promoter, similar to the endogenous MRP8 promoter [43]. In contrast, the CBX3 region of the A2UCOE remained free of CpG methylation for 21 days and, when placed next to the MRP8 promoter, partially prevented positional silencing [43]. This resembles the effect of A2UCOE orientation on the extent of CpG methylation at a SFFV promoter [41]. It is not yet known which feature(s) of the A2UCOE are responsible for this orientation-dependence, but it appears that the divergent promoters are not entirely equivalent.

An 8 kb A2UCOE fragment reduced DNA methylation of an adjacent CMV promoter in the majority of CHO-K1 clonal cell lines over 120 generations in culture [29]. However, one UCOE-containing clone showed substantial methylation similar to control levels, correlating with a ∼ 70 % reduction in transgene expression [29]. This shows that the ability of the UCOE to resist DNA methylation may depend on the genomic context of integration [29]. Despite this exception, the authors suggested that the A2UCOE can reduce transcriptional silencing by blocking encroachment of DNA methylation over the linked promoter [29].

**Fig. 3:**
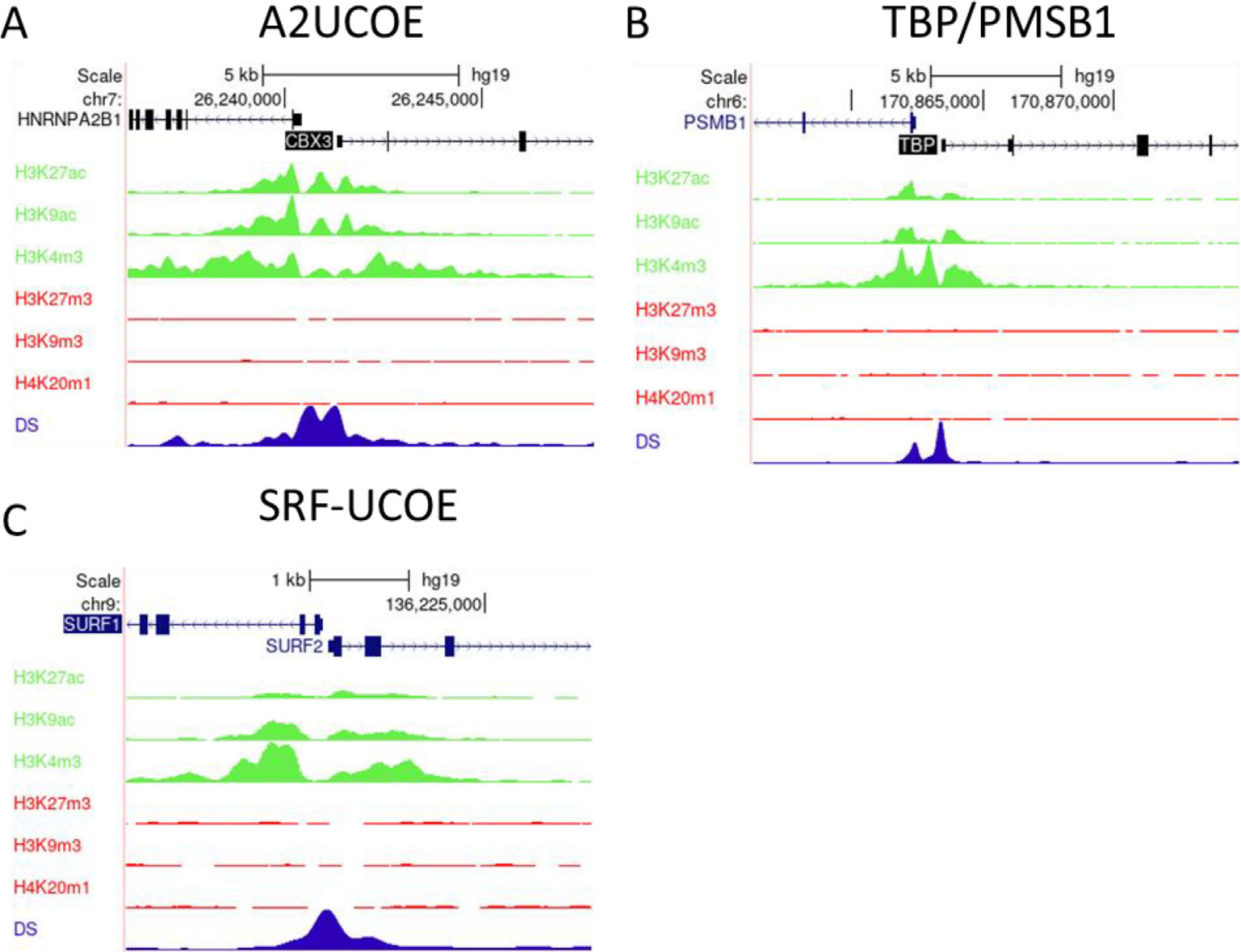
**UCOEs are characterised by peaks of euchromatic histone marks and depleted of heterochromatic marks in GM12878 cells.** Screenshots of UCSC genome browser (http://genome.ucsc.edu) showing group auto-scaled ENCODE ChIP-Seq peaks for active (green) and repressive (red) chromatin marks, and DNase I hypersensitivity (blue) at the (A) A2UCOE, (B) TBP/PSMB1, and (C) SRF-UCOE. (For interpretation of the references to colour in this figure legend, the reader is referred to the web version of this article.)

**Fig. 4:**
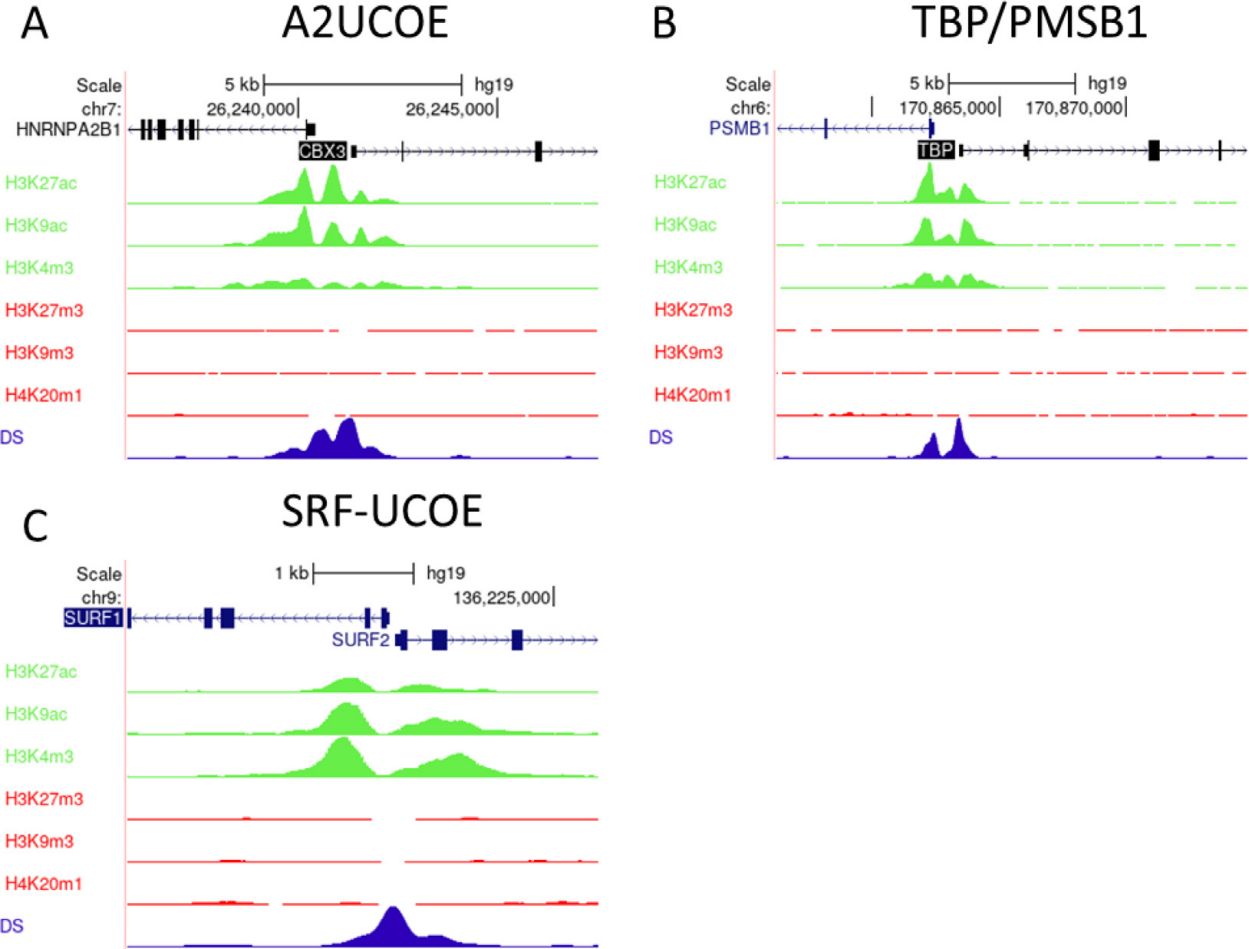
**UCOEs are characterised by peaks of euchromatic histone marks and depleted of heterochromatic marks in HeLa-S3 cells.** Screenshots of UCSC genome browser (http://genome.ucsc.edu) showing group auto-scaled ENCODE ChIP-Seq peaks for active (green) and repressive (red) chromatin marks, and DNase I hypersensitivity (blue) at the (A) A2UCOE, (B) TBP/PSMB1, and (C) SRF-UCOE. (For interpretation of the references to colour in this figure legend, the reader is referred to the web version of this article.)

**Fig. 5:**
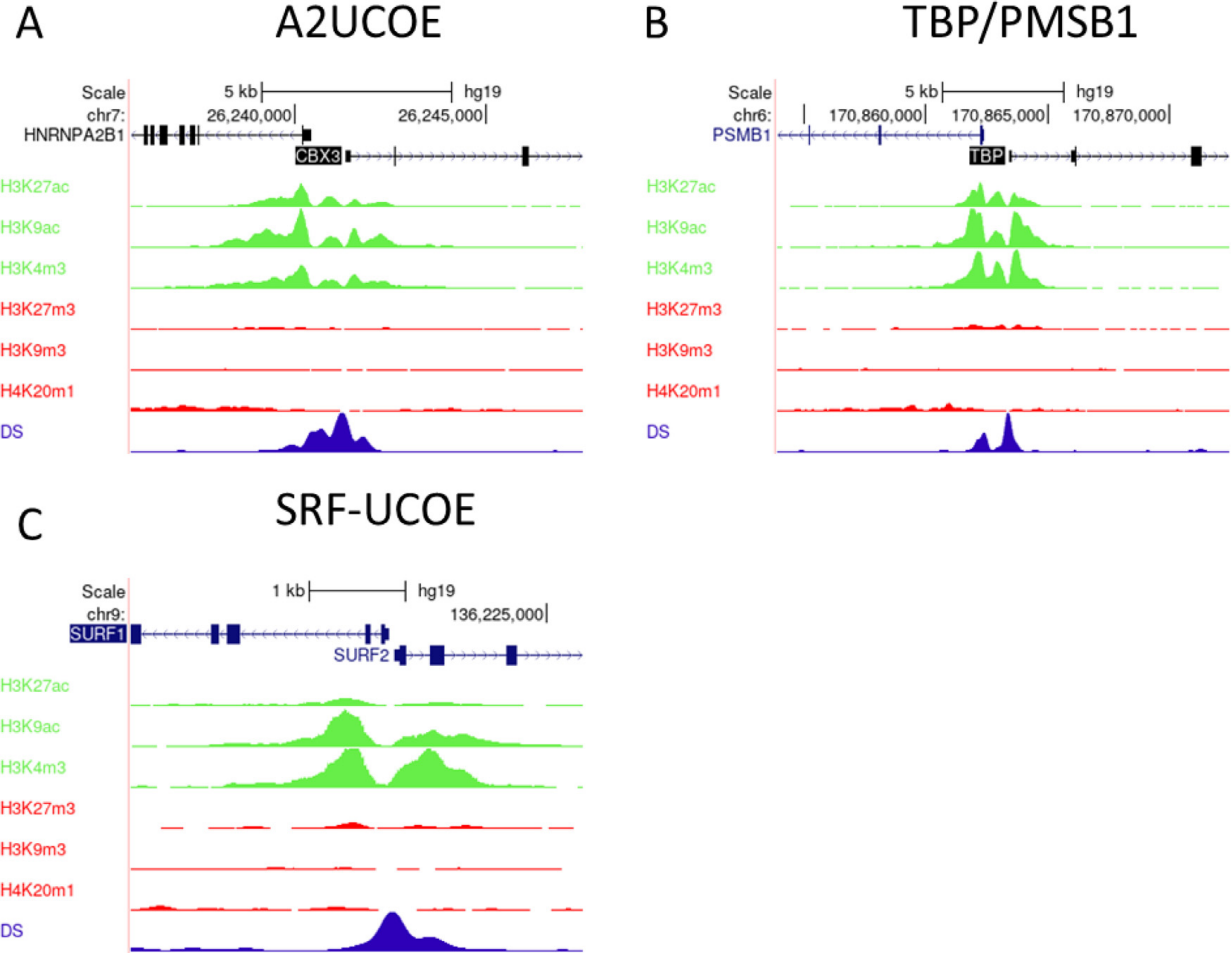
**UCOEs are characterised by peaks of euchromatic histone marks and depleted of heterochromatic marks in HepG2 cells.** Screenshots of UCSC genome browser (http://genome.ucsc.edu) showing group auto-scaled ENCODE ChIP-Seq peaks for active (green) and repressive (red) chromatin marks, and DNase I hypersensitivity (blue) at the (A) A2UCOE, (B) TBP/PSMB1, and (C) SRF-UCOE. (For interpretation of the references to colour in this figure legend, the reader is referred to the web version of this article.)

**Fig. 6:**
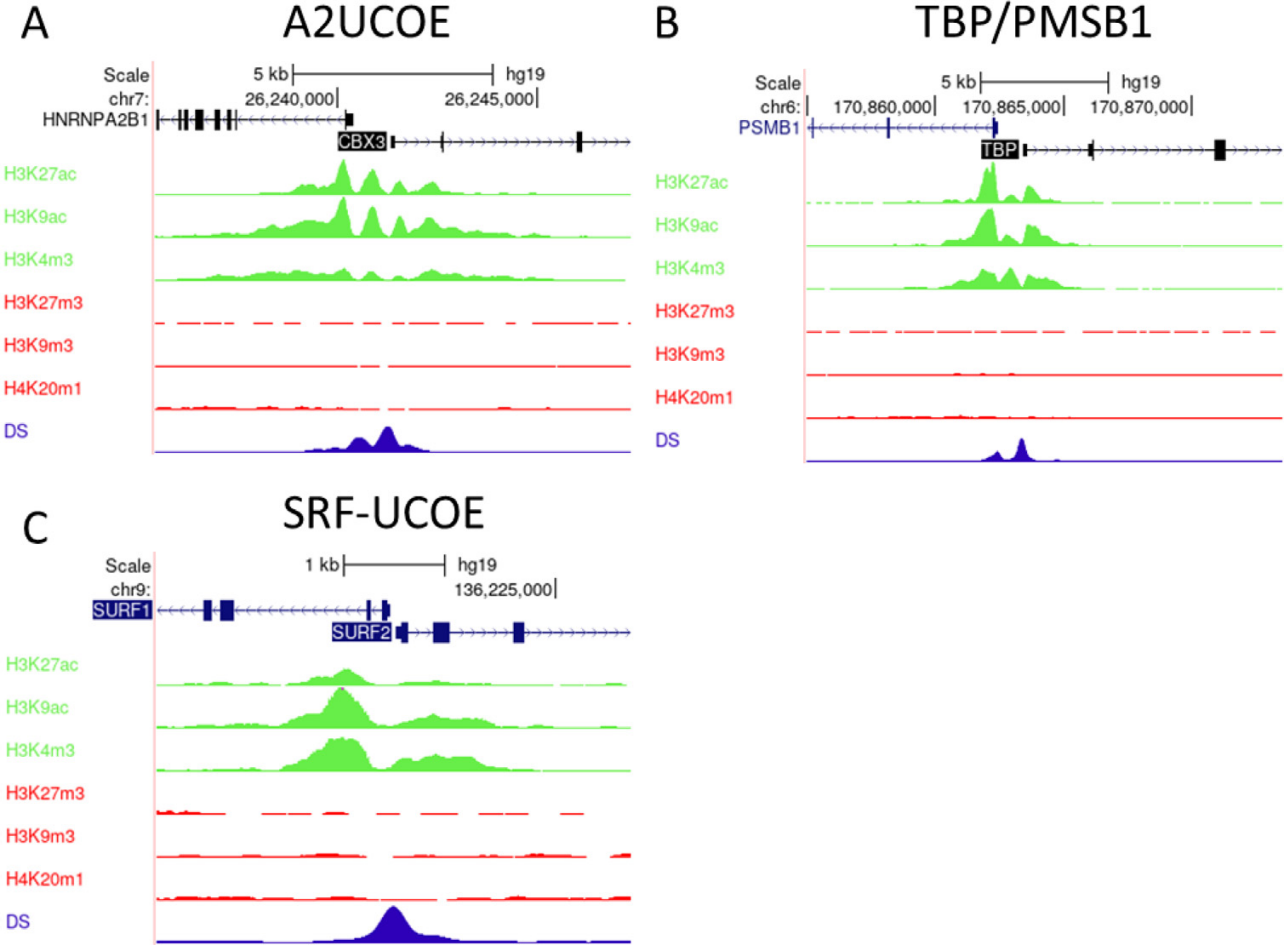
**UCOEs’ are characterised by peaks of euchromatic histone marks and depleted of heterochromatic marks in K562 cells.** Screenshots of UCSC genome browser (http://genome.ucsc.edu) showing group auto-scaled ENCODE ChIP-Seq peaks for active (green) and repressive (red) chromatin marks, and DNase I hypersensitivity (blue) at the (A) A2UCOE, (B) TBP/PSMB1, and (C) SRF-UCOE. (For interpretation of the references to colour in this figure legend, the reader is referred to the web version of this article.)

**Fig. 7:**
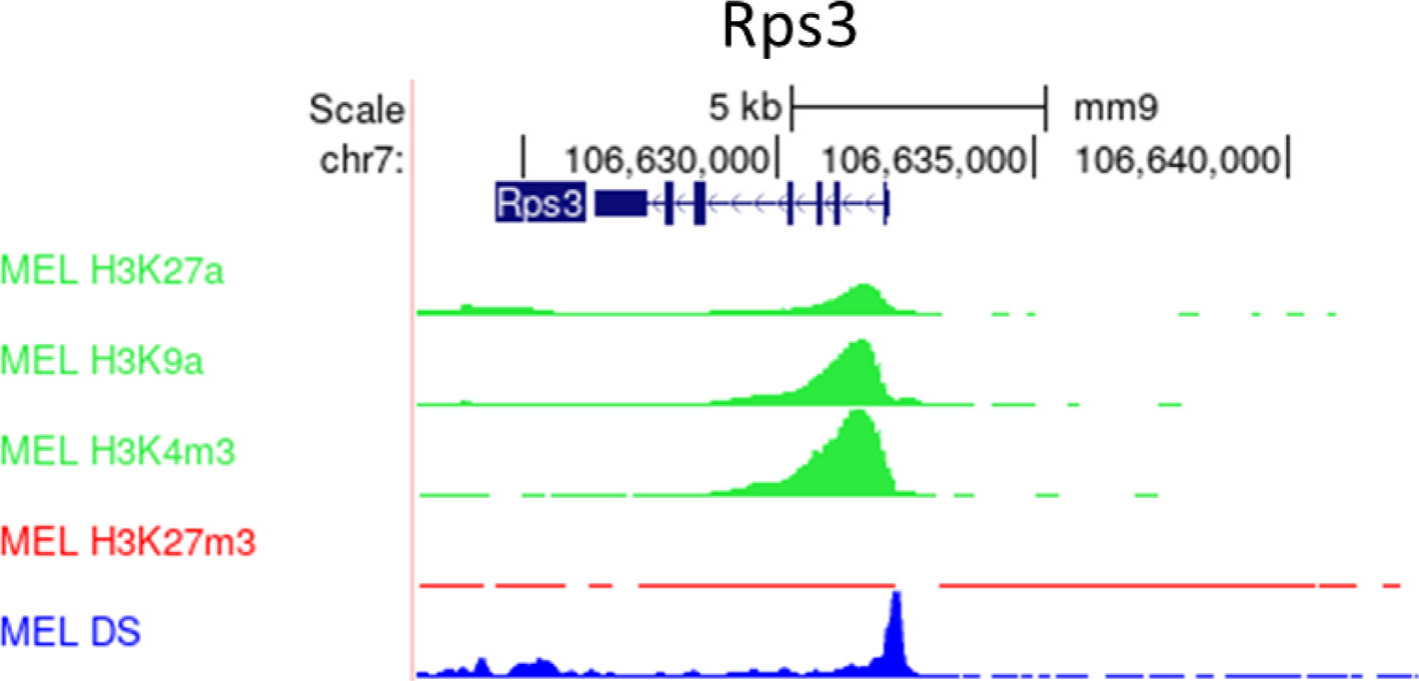
**The Rps3-UCOE is characterised by peaks of euchromatic histone marks and depleted of heterochromatic marks.** Screenshots of UCSC genome browser (http://genome.ucsc.edu) showing group auto-scaled ENCODE ChIP-Seq peaks for active (green) and repressive (red) chromatin marks, and DNase I hypersensitivity (blue) in MEL cells. (For interpretation of the references to colour in this figure legend, the reader is referred to the web version of this article.)

To date, mechanistic research into how UCOEs negate silencing has been limited to the A2UCOE. However, a large body of ChIP-Seq datasets detailing chromatin environment has become publicly available in recent years, especially for the human genome. Such data provide an opportunity to analyse chromatin modifications surrounding endogenous UCOE loci in multiple cell lines, including the lymphoblastoid cell line GM12878 (Fig. 3), HeLa cervical carcinoma cells (Fig. 4), HepG2 hepatocellular carcinoma cells (Fig. 5), and K562 erythroleukemia cells (Fig. 6). Bioinformatic analysis of ENCODE ChIP-seq datasets within the UCSC genome browser for these unrelated human cell types revealed that in each case the TBP/PSMB1 and SURF1/SURF2 loci show similar epigenetic patterns to the A2UCOE, with peaks of euchromatic marks H3K27ac, H3K9ac and H3K4me3 that are associated with active expression, and relative depletion of repressive marks H3K27me3, H3K9me3 and H4K20me1 [44], [45]. Furthermore, the loci are hypersensitive to DNase I, a standard test for accessible chromatin. These same properties are observed for the mouse Rsp3 locus in murine erythroleukemia (MEL) cells (Fig. 7). Thus, the four UCOE loci in their native contexts show similar epigenetic properties across species and cell types.

All three human UCOEs have an extensive CpG island which includes the dual promoter regions (Fig. 1). The A2UCOE and SRF-UCOE have a single CpG island, which incorporates a high percentage of C or G nucleotides (61.5 % and 59.6 % respectively) and a high density of CpG dinucleotides (18.2 % and 20.4 % respectively) [46]. In contrast, the TBP/PSMB1 UCOE has two distinct CpG islands that are 840 bp and 610pb in length and are located, respectively, over the TBP and PSMB1 transcriptional start sites. Together, these methylation-free regions include 57 % C and G nucleotides with 7 % CpG dinucleotides [32]. It is yet to be determined if the TBP/PSMB1 and SRF-UCOE confer stable expression by negating DNA methylation, but the high content of unmethylated CpG may open the chromatin and promote active transcription, similar to the A2UCOE [33].

Although the organisational similarities and common epigenetic signatures of the UCOEs point toward shared mechanisms of action, the defining characteristics remain uncertain. The Rps3 UCOE demonstrates that a bidirectional promoter is not essential for activity. The four UCOEs all have CpG islands, but these are found in 60–70 % of vertebrate promoters [47] and an isolated CpG island was insufficient to augment stable expression [36]. The modification state of histones at UCOEs may be especially important for the stability of expression, since DNA methylation changes relatively little during long term culture of CHO cells [48], [49]. The histone acetylation and DNase I hypersensitivity are likely to be generated by sequence-specific transcription factors that recruit acetyltransferases and chromatin remodelling complexes, but the identities of these have yet to be established. Apart from UCOEs, many promoters contain DNase I-accessible CpG islands with acetylated histones. Are additional features required or are there thousands of loci with unappreciated UCOE potential?

## 4 Use of UCOEs in biomanufacturing

Biopharmaceutical manufacturing commonly involves the random integration of transgene DNA into the genome of a host cell, such as CHO. Expression from different integration sites is highly variable due to differing epigenetic environments. To control for population heterogeneity and production instability caused by varying epigenetic environments, vectors can be engineered to include gene regulatory elements, such as UCOEs [30].

The first use of a UCOE to improve recombinant protein titre was reported in 2002 [50], when an 8 kb A2UCOE inserted upstream of a hCMV promoter led to the production of 0.2 g/L of a monoclonal antibody (mAb) from CHO cells. A subsequent study showed that the inclusion of the A2UCOE increased the percentage of high-expressing cells, with 90 % of recombinant cells producing > 0.5 μg/ml of erythropoietin (EPO), compared to 5 % of cells in non-UCOE lines, and high expression was maintained over 174 generations [35]. Although these early studies highlighted benefits of using UCOEs to reduce population heterogeneity and stabilise transgene expression, they were limited by the continuous use of selection drugs throughout the stability studies [35].

### 4.1 UCOEs increase production stability during long-term culture

UCOEs’ ability to confer stable expression during long-term culture has been demonstrated in multiple industrially-relevant cell lines, including several lineages of CHO cells [42], [51], [52], [53]. Boscolo and colleagues showed that a 4 kb fragment of the A2UCOE upstream of the hCMV promoter increased not only the number of expressing CHO-S clones, but also the stability of expression, with no detectable decrease in scFv fusion protein level after 52 days without selection [51]. Similarly, chimeric mAb expression from a CMV promoter in CHO-K1 cells decreased only 9.5 % over 8 weeks in the presence of an upstream 1.5 kb A2UCOE fragment, against 50 % without a UCOE [53]. The same A2UCOE fragment also benefits a sleeping beauty transposon system, maintaining transgene expression in CHO-K1 cells over seven weeks of continuous culture, independently of orientation [52]. The ability of this shorter core UCOE fragment to ameliorate silencing could benefit vectors with limited packaging size, especially relative to other insulators that need to be incorporated on both sides of the GOI.

UCOEs have also been tested following site-specific integration into a predetermined genomic site. The Rps3 UCOE and 1.5 kb A2UCOE flanking an antibody light chain transgene stabilised its expression over six weeks of continuous culture at two distinct chromosomal locations [54]. Furthermore, GOI expression was highly homogeneous between individual cells, suggesting that UCOEs allow replicable expression from a single gene copy. For greater industrial relevance, it will be necessary to test longer culture periods.

Gene amplification methods can be valuable for producing high-expressing clones [55]. An example involves subjecting CHO-DG44 cells, which are deficient for dihydrofolate reductase (DHFR), to increasing amounts of methotrexate (MTX) after transfection with a vector encoding a DHFR gene along with a GOI [56], [57]. The MTX selects for amplification of the exogenous DHFR gene, which leads to amplification of the adjacent GOI. When tested in this context, an 8 kb A2UCOE fragment was unable to improve the stability of production of EPO or eGFP mRNA or protein over 77–80 days of culture, in the absence of selection pressure [58], [59]. Perhaps in this context genomic instability associated with gene amplification dominates over epigenetic effects. As the authors pointed out, UCOEs were still advantageous under these conditions, as they promoted favourable growth characteristics and reduced population heterogeneity, an effect attributed to fewer chromosomal rearrangements following MTX treatment in UCOE-containing cell lines [58], [59], [60]. Similar results were reported in CHO-DG44 cells producing recombinant anti-TNFa; a 1.5 kb A2UCOE fragment increased the number of high-expressing clones, but failed to improve the stability of expression over 16 weeks of culture in the absence of selective pressure; in this case, instability was shown to correlate with a significant loss of transgene copies [61].

### 4.2 UCOEs enhance recombinant protein titre

The ability of UCOEs to increase recombinant protein titre irrespective of stability has been widely reported. Inclusion of a 1.5 kb A2UCOE fragment doubled production of an anti-IL-2Ra mAb from 370 μg/L to 746 μg/L in a pool of CHO-K1 cells without significant changes in copy number [53]. Furthermore, the top UCOE-containing clones produced ∼ 3-fold more mAb on average than the non-UCOE equivalents. The same A2UCOE fragment boosted the titre of anti-TNFa in CHO-DG44 cells by 2.1-fold following 7 days of culture [61]. Although promising, these data were limited to small scale production protocols and have yet to be demonstrated in bioreactors.

Expression of adalimumab from a monocistronic pFUSE expression vector was significantly enhanced by placing the Rps3 UCOE upstream of a CMV promoter [62]. When the A2UCOE was also included, the double UCOE construct increased the percentage of high-expressing CHO-K1 cells, with 40.5 % of cells producing > 50 μg/ml, whereas the control expression vector failed to exceed 10 μg/ml [62]. When tested with a ClonePix robotic clonal selection system, the 3.2 kb Rps3 UCOE or the 8 kb A2UCOE boosted IgG1 mAb expression driven by a hCMV promoter in suspension-adapted CHO-K1 cells; however, the effect was dependent on the transgene, with only moderate increases in IgG4 expression [63]. Perhaps a downstream step, such as translation or secretion, was limiting for IgG4 production in this example.

The use of UCOEs to increase recombinant protein production is not limited to full-sized mAbs. A 4 kb A2UCOE fragment upstream of a hCMV promoter increased yield of anti-CD55 and anti-C5 scFv-Fc fusion proteins in a CELLine flask system [51]. An 8 kb A2UCOE fragment increased the volumetric production per gene copy of EPO in CHO-DG44 cells, even in combination with the MTX amplification system [59].

### 4.3 UCOEs’ effects are influenced by the promoter

To add further complexity, the efficiency of UCOEs is highly dependent on the UCOE-promoter combination. Nair and colleagues investigated the effect of the 3.2 kb Rps3 UCOE or the 1.5 kb A2UCOE on expression of a large protein, B domain-deleted factor VIII (BDD-VIII), driven by 5 different promoters in stably transfected baby hamster kidney 21 (BHK21) cells [64]. The 1.5 kb A2UCOE fragment outperformed the Rsp3 UCOE when used in combination with the mCMV, rCMV or Ubc promoters, increasing specific productivity by ∼ 6-fold, ∼1.3-fold and ∼ 5-fold, respectively, but these beneficial effects of the A2UCOE were lost when combined with the gCMV or SRa promoters [64]. Furthermore, promoter-UCOE combinations behaved differently in other cell lines [64], illustrating the importance of optimising UCOE vectors for a given cell line. Comparison of promoter-UCOE combinations in P19 cells found that A2UCOE improved stability when linked to three promoters, but the SRF-UCOE performed better with a fourth [36]. This could indicate promoter-dependent variations in epigenetic silencing that respond differently to alternative UCOEs.

The effect of UCOEs has also been tested in cells harbouring separate light chain and heavy chain antibody vectors; the highest titre was obtained when A2UCOE was placed only in the heavy chain vector, increasing the titre 5- to 8-fold over alternative combinations [65]. The authors reasoned that antibody production is limited by availability of the heavy chain.

Increasing the number of UCOEs relative to the GOI can boost expression. Thus, two UCOE copies allowed higher yields of anti-TNFa than a single UCOE copy, dependent on the choice of promoter [66]. Furthermore, three copies of the A2UCOE increased titre 6.5-fold whereas two, or one copies were ∼ 2-fold and ∼ 3-fold less effective, respectively [29]. These results provide clear evidence that the effect of the A2UCOE is additive.

## 5 Summary and outlook

The past 20 years have witnessed impressive progress in the development of recombinant cell lines and biomanufacturing technologies, with far-reaching implications for the availability of pharmaceuticals. Here, we have assessed the UCOE class of DNA elements for their ability to promote enhanced and sustained transgene expression in cell lines that are commonly used for biomanufacturing. There is evidence that UCOEs encourage a favourable epigenetic environment that is depleted of DNA methylation and repressive histone marks. Incorporation of UCOEs into vectors can be advantageous, generating a more homogeneous population of expressing cells and a greater number of high-expressing clones. As such, UCOE-based gene expression systems offer an attractive strategy to reduce the time and cost associated with upstream biomanufacturing stages. The A2UCOE is the most extensively studied and epigenetic characterisation has been limited to this UCOE. By mining published ChIP-Seq datasets bioinformatically, we have found that all four known UCOEs possess similar patterns of histone modifications, suggesting shared molecular mechanisms of action (Fig. 3, Fig. 4, Fig. 5, Fig. 6, Fig. 7). Further epigenomic characterisation may help establish the optimal combination of histone marks to facilitate development of synthetic UCOEs that effectively stabilise high transgene expression [15], [48], [49]. Both the A2UCOE and the Rsp3 UCOE have demonstrated ability to improve transgene expression within multiple cell systems, including both viral and non-viral vectors, suggesting potential application for gene therapies. The best UCOE-promoter combination should be determined in each context, to ensure maximal titre and stability, especially after gene amplification. A combinatorial approach, coupling different genetic elements, could reduce instability of expression following gene amplification, while maintaining the high productivity induced by the A2UCOE. Widespread inclusion of UCOEs in biomanufacturing vectors has potential to improve the epigenetic environment of integrated transgenes and thereby stimulate their expression during long-term culture.

## CRediT authorship contribution statement

**Rebecca E. Sizer:** Data curation, Investigation, Formal analysis, Writing – original draft. **Robert J. White:** Conceptualization, Supervision, Writing – review & editing.

## Declaration of Competing Interest

The authors declare that they have no known competing financial interests or personal relationships that could have appeared to influence the work reported in this paper.
